# The Retina of Osteopontin deficient Mice in Aging

**DOI:** 10.1007/s12035-017-0734-9

**Published:** 2017-09-02

**Authors:** Noelia Ruzafa, Xandra Pereiro, Patricia Aspichueta, Javier Araiz, Elena Vecino

**Affiliations:** 1 Department of Cell Biology and Histology, University of Basque Country, www.ehu.es/GOBE, Experimental Ophtalmo-Biology Group UPV/EHU, Vizcaya, Spain; 2 Biocruces Research Institute, Barakaldo, Spain; 3 Department of Physiology, University of the Basque Country UPV/EHU, Vizcaya, Spain; 4 Department of Ophthalmology, University of the Basque Country UPV/EHU, Vizcaya, Spain

**Keywords:** Retina, Osteopontin, Retinal ganglion cells, Glia, Astrocytes, Müller cells, Microglia

## Abstract

Osteopontin (OPN) is a secreted glycosylated phosphoprotein that influences cell survival, inflammation, migration, and homeostasis after injury. As the role of OPN in the retina remains unclear, this study issue was addressed by aiming to study how the absence of OPN in knock-out mice affects the retina and the influence of age on these effects. The study focused on retinal ganglion cells (RGCs) and glial cells (astrocytes, Müller cells, and resident microglia) in 3- and 20-month-old mice. The number of RGCs in the retina was quantified and the area occupied by astrocytes was measured. In addition, the morphology of Müller cells and microglia was examined in retinal sections. The deficiency in OPN reduces RGC density by 25.09% at 3 months of age and by 60.37% at 20 months of age. The astrocyte area was also reduced by 51.01% in 3-month-old mice and by 57.84% at 20 months of age, although Müller glia and microglia did not seem to be affected by the lack of OPN. This study demonstrates the influence of OPN on astrocytes and RGCs, whereby the absence of OPN in the retina diminishes the area occupied by astrocytes and produces a secondary reduction in the number of RGCs. Accordingly, OPN could be a target to develop therapies to combat neurodegenerative diseases and astrocytes may represent a key mediator of such effects.

## Introduction

Osteopontin (OPN) is a secreted glycosylated phosphoprotein encoded by the *spp1* gene [1, 2] that has an arginine-glycine-aspartic acid cell-binding sequence [3, 4]. OPN is a cytokine that binds to integrins and CD44 variants on the cell surface [5], integrins fulfilling many different functions in cells that influence: cell survival and apoptosis, inflammation, microcalcification, cell attachment and migration, intracellular signaling, chemotaxis [6, 7], the maintenance of homeostasis after an injury [8], and the modulation of neuronal regeneration following injury [7]. OPN can be found in the nervous system, in the developing and adult rodent brain, and neurons in the olfactory bulb, retina, striatum, and brainstem-produced OPN [9–11]. Moreover, while OPN may be only weakly expressed under physiological conditions, it may augment during inflammation and in neurodegenerative diseases [1, 2, 7, 12].

In the retina, OPN is expressed by retinal ganglion cells (RGCs) [13], the neurons that relay visual signals to the brain [14]. RGCs are also the retinal cells most vulnerable to ischemic and excitotoxic insults [15], which upregulate OPN expression [13] to possibly afford protection against death, as occurs in experimental glaucoma [16, 17]. The retinal glia, astrocytes, microglia, and Müller cells maintain homeostasis within the retina, where they provide structural support and influence metabolism, phagocytosis of neuronal debris, immune responses, and other activities [18]. Reactive astrocytes express OPN following different types of brain insult [19–21], and it has also been associated with the microglia that fulfill macrophagic functions [18]. As occurs in astrocytes, OPN is upregulated in activated microglia after CNS damage [22–27], suggesting that it may play a key role in the pathogenesis of neuroinflammation [20]. OPN binds to the integrin αvβ3 receptor, which means it may act as a chemoattractant in recruiting astrocytes and microglia during the formation of the glial scar following ischemic injury [20, 23, 24, 28, 29]. Indeed, OPN may be involved in glial activation, cell repair, glial and macrophage migration, and matrix remodeling by reactive astrocytes [20, 24, 28, 29]. On this basis, the effect of OPN on retinal astrocytes and microglia is worthy of study. Moreover, OPN is found in the secretome of Müller cells [30, 31], and as a result, their morphology has been studied to understand how OPN affects these retinal glial cells.

Aging is the main risk factor for neurodegenerative diseases and OPN has age-dependent neuroprotective effects [32]. Several modifications occurs in the normal retina with aging, which include RGC loss, stronger GFAP (glial fibrillary acidic protein) expression by astrocytes, an increase in cytoplasmic organelles [33–35], and the acquisition of an activated phenotype by microglial cells [36]. Thus, it is important to compare how the absence of OPN affects the retina in young (3-month-old) and old (20-month-old) mice.

The aim of this study was to investigate the effect of OPN deficiency in the retina using an OPN knock-out mouse, focusing on RGCs, astrocytes, microglia, and Müller cells. In addition, as the influencing of aging on OPN function is unclear, the effects of this deficiency were studied in animals of different ages.

## Materials and Methods

### Animals

Female knock-out (B6.129S6(Cg)-*Spp1* ^tm1Blh^/J) and C57BL/6J mice aged 3 months (*n* = 3 wild type and *n* = 3 knock-out) and 20 months old (*n* = 4 wild type and *n* = 4 knock-out) were used in these experiments (The Jackson Laboratory, Bar Harbor, ME, USA). The animals had free access to food and water, and they were kept at a constant temperature of 21 °C on a 12 h light-dark cycle. All procedures were carried out in adherence to the ARVO Statement for the Use of Animals in Ophthalmic and Vision Research.

### Tissue Collection

Animals were sacrificed by cervical dislocation and their eyes were enucleated. The cornea, crystalline lens, and the vitreous were removed, and the retina was carefully extracted. The retina was immediately fixed for 5 h in 4% paraformaldehyde (PFA) prepared in 0.1-M phosphate buffer (pH 7.4) and then extended on filter paper (Millipore, Madrid, Spain). To obtain sections, the eyes were extracted and immediately fixed overnight in 4% PFA. They were then cryoprotected for 24 h in 30% sucrose in 0.1-M phosphate buffer at 4 °C and embedded in OCT (optimal cutting temperature) medium. Cryosections (14-μm thick) were obtained and stored at − 20 °C.

### Immunochemistry

Whole mount retinas were immunostained as described previously [37], with some minor modifications. The flat fixed retinas were washed in phosphate-buffered saline (PBS, pH 7.4), and they were blocked by incubating them overnight with shaking at 4 °C in a solution of PBS-TX-100-BSA (0.25% Triton-X 100 and 1% bovine serum albumin in PBS). The retinas were then incubated for 1 day at 4 °C with the primary antibodies diluted in PBS-TX-100-BSA: an anti-RBPMS (RNA-binding protein with multiple splicing) guinea pig antibody (1:4000 PhosphoSolutions, Aurora, USA) to detect RGCs; and an anti-GFAP mouse antibody (1:1000 Sigma, Steinheim, Germany) to detect astrocytes. Subsequently, the retinas were washed three times in PBS for 15 min and antibody binding was detected over 5 h at room temperature with shaking using secondary antibodies diluted 1:1000 in PBS-BSA (1%): an Alexa Fluor 555 conjugated goat anti-guinea pig antibody (Invitrogen, Eugene, OR, USA) and an Alexa Fluor 488 conjugated goat anti-mouse antibody (Invitrogen). Finally, the retinas were washed three times for 10 min in PBS, flat mounted onto slides in PBS:glycerol (1:1), and coverslipped.

Microglia and Müller cells were immunostained in cryostat sections of the eye, as described previously [38]. The sections were washed twice with PBS-TX-100 for 10 min, and they were then incubated overnight with a primary rabbit anti-Iba1 antibody (1:2000; Wako, Osaka, Japan) to detect microglia and a rabbit antibody against glutamine synthetase (1:10,000; Abcam, Cambridge, UK) to detect Müller cells. The RGCs were also labeled with the anti-RBPMS guinea pig antibody. After two washes with PBS, antibody binding was detected for 1 h with Alexa Fluor 555 or 488 goat anti-rabbit or anti-guinea pig secondary antibodies (Invitrogen) diluted 1:1000 in PBS-BSA (1%). The sections were washed twice with PBS for 10 min and mounted with a coverslip with PBS:glycerol (1:1).

### Image Capture

Images were acquired with a digital camera (Zeiss Axiocam MRM, Zeiss, Jena, Germany) coupled to an epifluorescence microscope (Zeiss) using the Zen software (Zeiss). For whole-mount retinas, a mosaic of the entire retina was generated using the 555 and 488 filters with a ×10 objective. The area of the mosaic was defined in overlapping micrographs of a defined area of the retina obtained automatically, and once the mosaic was defined, the contour of the retina was measured and the retinal surface area was calculated.

### RGC Quantification

The number of RGCs was counted semi-automatically using Zen software (Zeiss) and the RGC density (mean ± standard error) was compared between the wild type and OPN knock-out retinas using a Student’s *T* test. In addition, a Mann-Whitney *U* test was used to verify the differences between groups. These statistical analyses were carried out using IBM SPSS Statistics software v. 21.0. For both tests, the minimum value of significant differences was defined as *p* < 0.05.

### Astrocyte Morphology

The morphology of astrocytes was analyzed using the ImageJ image processing and analysis software developed at the National Institutes of Health (NIH, version 1.49). The region formed by the colored pixels (labeled with the antibody against GFAP) was selected using the “threshold color” tool, and the surface area of the selected astrocytes was measured. We calculated the proportion of the retinal surface occupied by astrocytes and we compared this in the most central part of the retina (1-mm diameter) of control and OPN knock-out mice, taking the optic nerve as the center yet disregarding this structure. At the optic nerve, the morphology of the astrocyte’s branches impedes their quantification due to their complexity.

Statistical analyses were again carried out using IBM SPSS Statistics software v. 21.0. The average of the percentages and the standard errors were calculated and compared for the wild type and OPN knock-out retinas using a Student *T* test, and it was supplemented with Mann-Whitney *U* test to verify the differences between the groups. The minimum value for significant differences for both tests was defined as *p* < 0.05.

## Results

**Fig. 1:**
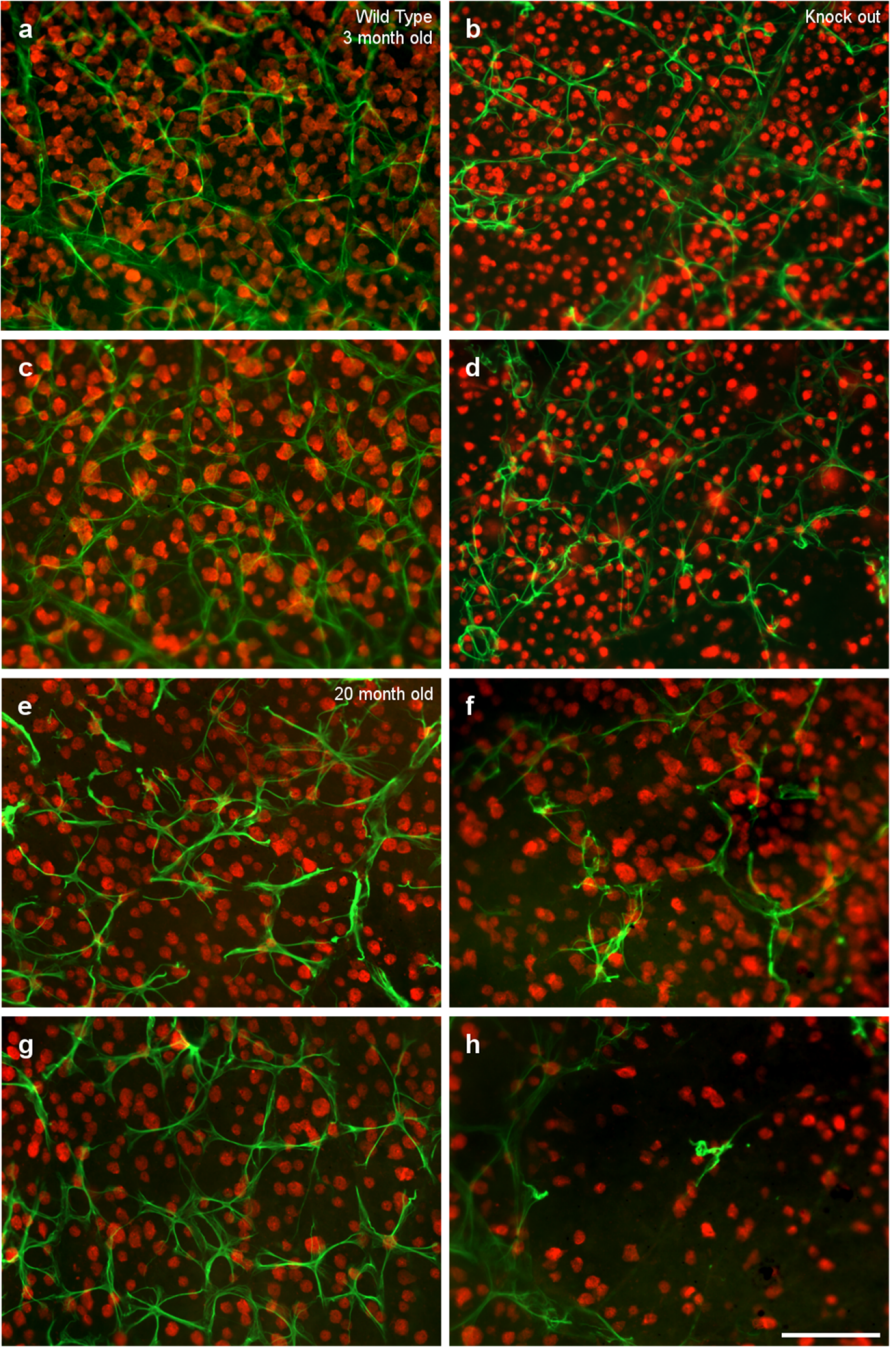
Flat mount retinas. Images of whole mount retinas from wild type (a, c, e, g) and OPN knock-out (b, d, f, h) mice of two different ages: 3 (a, b, c, d) and 20 months of age (e, f, g, h). The retinas were labeled with an antibody against GFAP (green) to identify astrocytes and an antibody against RBPMS (red) to label RGCs. The pictures were taken at a distance of 1 (a, b, e, f) and 2.5 mm (c, d, g, h) from the optic nerve. Scale bar = 100 μm

The RGCs and astrocytes in the retina of 3- and 20-month-old wild type and OPN knock-out mice were analyzed by labeling them with an antibody against RBPMS or GFAP, respectively (Fig. 1). There were some regions in the retina of the OPN knock-out mice where the cell density was very low, particularly in the peripheral areas, 2.5 mm from the optic nerve. In these regions, the low density of RGCs and astrocytes was further exaggerated with age.

**Fig. 2:**
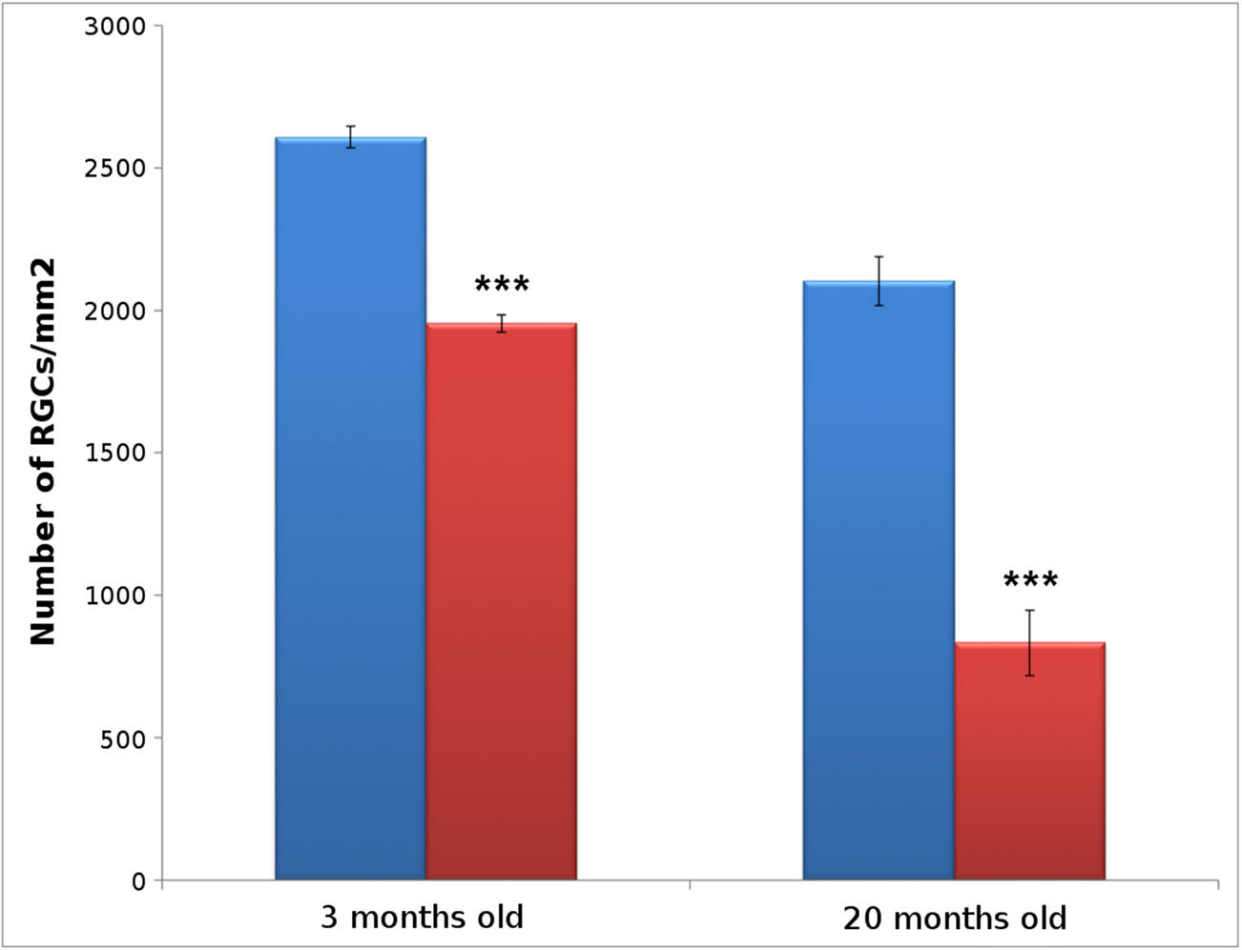
Retinal ganglion cell analysis. Histogram representing the number of RGCs (RGCs/mm^2^) in wild type (blue) and OPN knock-out (red) mice at two different ages: 3- and 20-months-old: *** *p* < 0.001

The number of RGCs labeled with RBPMS in the entire retina and the area of each retina was evaluated and their density calculated (RGCs/mm^2^). In 3-month-old wild type mice, the average RGC density (2607.15 ± 38.36 RGCs/mm^2^) was greater than in the OPN knock-out mice (1953.37 ± 29.75 RGCs/mm^2^), as was also evident in 20-month-old animals (wild type 2101.86 ± 84.73 RGCs/mm^2^; OPN knock-out mice 832.77 ± 114.28 RGCs/mm^2^). Thus, the OPN deficiency triggered a mean reduction in RGC density of 25.09% at 3 months of age (*p* < 0.001), which was further reduced to 60.37% at 20 months of age (*p* < 0.001, Fig. 2).

In the retinas of wild type mice, astrocytes form a network that covers the whole surface of the retina, surrounding the vessels, and they are connected through their stellar morphology. By contrast, OPN knock-out mice seem to have fewer astrocytes and there are larger gaps between them, with less linear extensions than in the controls. The smaller number of astrocytes, with shorter branches and fewer processes, was suggestive of astroglial atrophy. This effect is more severe in 20-month-old mice than in 3-month-old mice, with larger areas lacking astrocytes and some cells that have lost their stellate morphology (Fig. 1e–h). It is important to note that there are more areas without astrocytes and a lower density of RGCs in the peripheral regions of the retina in OPN knock-out mice (Fig. 1d and h), relative to the regions closer to the optic nerve (Fig. 1b and f). These differences were found in both 3-month-old and 20-month-old mice.

**Fig. 3:**
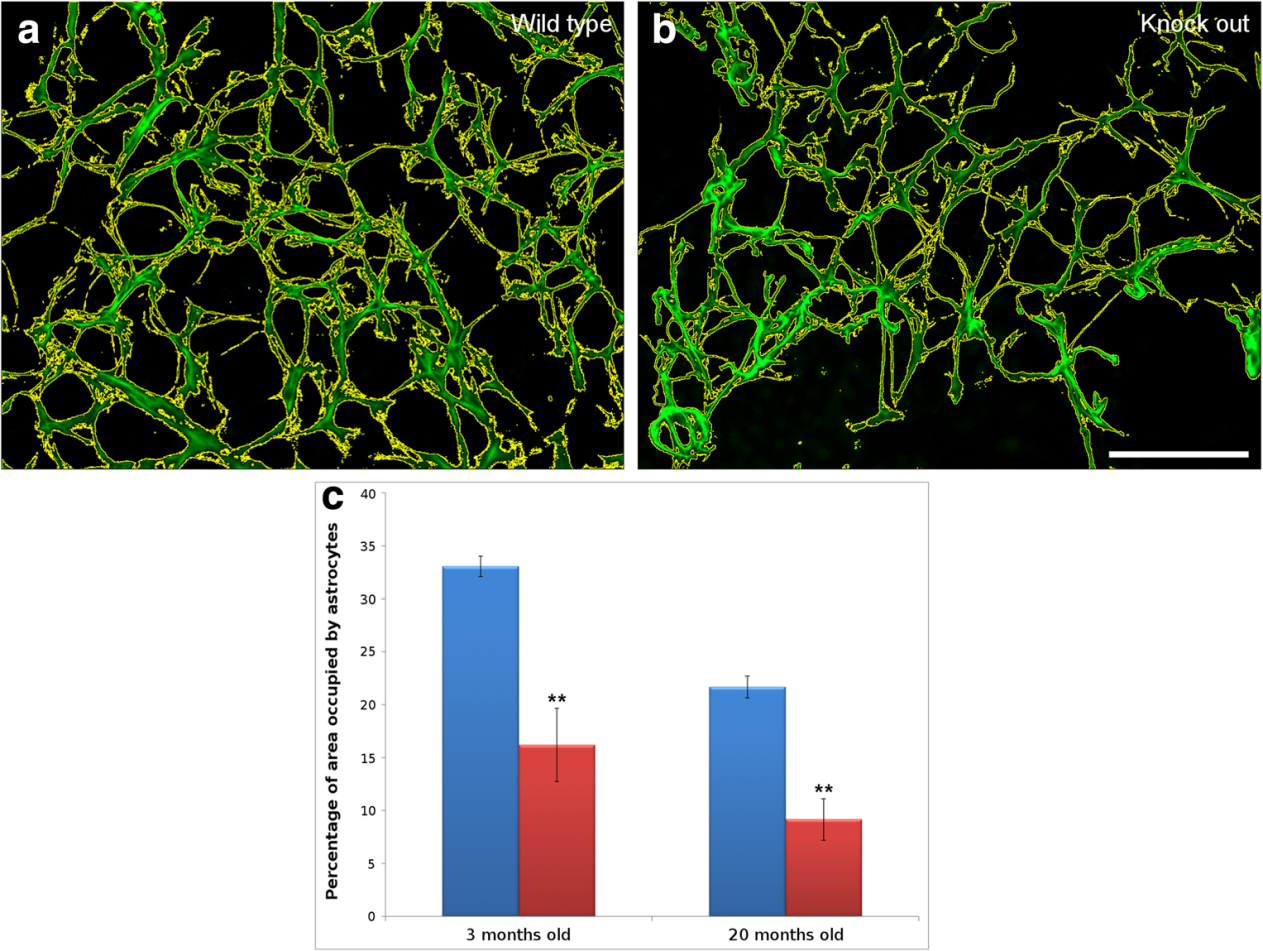
Astrocyte analysis. Measurement of the area occupied by the astrocytes labeled with an antibody against GFAP (green). The area defined by the yellow line was measured automatically. The astrocytes were compared between wild type (a) and OPN knock-out (b) mice. Astrocyte density was measured as the area occupied by astrocytes, and it was represented graphically in wild type (blue) and OPN knock-out (red) mice at 3 and 20 months of age (c). Scale bar = 100 μm: ** *p* < 0.01

When the area occupied by astrocytes was quantified, the average area occupied by astrocytes in 3-month-old wild type mice (33.05% ± 0.95) was greater than in the OPN knock-out mice (16.19% ± 3.47). Thus, the absence of OPN induces a 51.01% reduction in astrocyte density (*p* < 0.01). In 20-month-old mice, the proportion of the wild type retina occupied by astrocytes (21.67% ± 1.02) was still 57.84% higher than in the OPN knock-out mice (9.14% ± 1.95), confirming that the absence of OPN reduces the astrocyte density in the retina (*p* < 0.01, Fig. 3). Furthermore, the number of RGCs and the area covered by astrocytes in the 3-month-old OPN knock-out mice was very similar to the same parameters in the 20-month-old wild type mice, with no significant differences between these two ages.

**Fig. 4:**
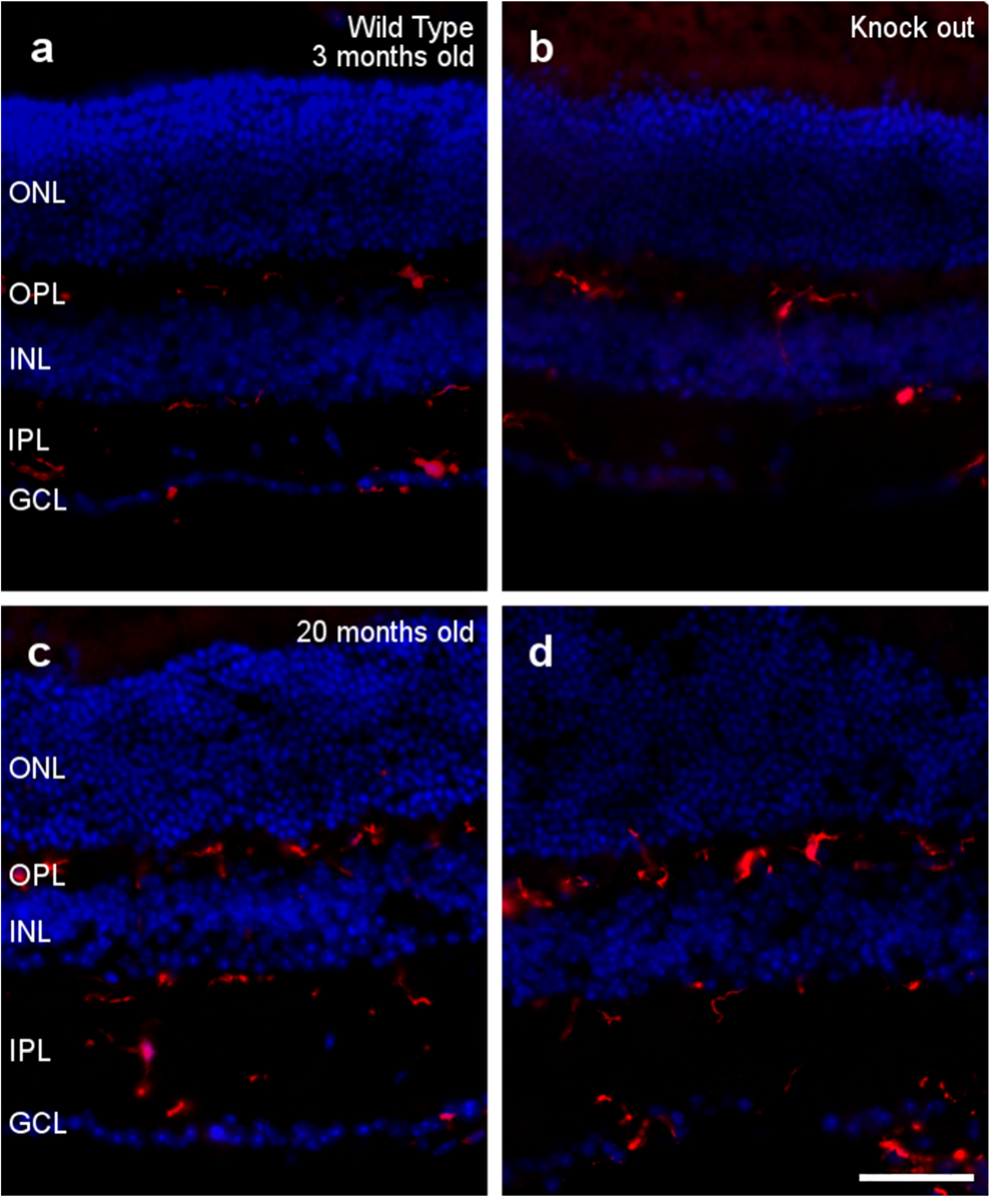
Microglial analysis. Retinal sections from 3- (a, b) and 20-month-old (c, d) wild type (a, c) and OPN knock-out (b, d) mice, in which the nuclei are labeled with DAPI (blue) and the microglia are labeled with an antibody against Iba1 (red): ONL, outer nuclear layer; OPL, outer plexiform layer; INL, inner nuclear layer; IPL, inner nuclear layer; GCL, ganglion cell layer. Scale bar = 50 μm

Interestingly, no signs of microglia activation were detected in the OPN knock-out retinas at 3 or 20 months of age (Fig. 4). The morphology of the microglia is similar in the presence and absence of OPN, and these cells do not seem to adopt an amoeboid aspect and they do not undergo morphological changes, such as a thickening and retraction of branches. Moreover, microglial cells are located at the inner part of the retina in all cases, meaning that they are not active as migration to the subretinal space is a sign of activation.

**Fig. 5:**
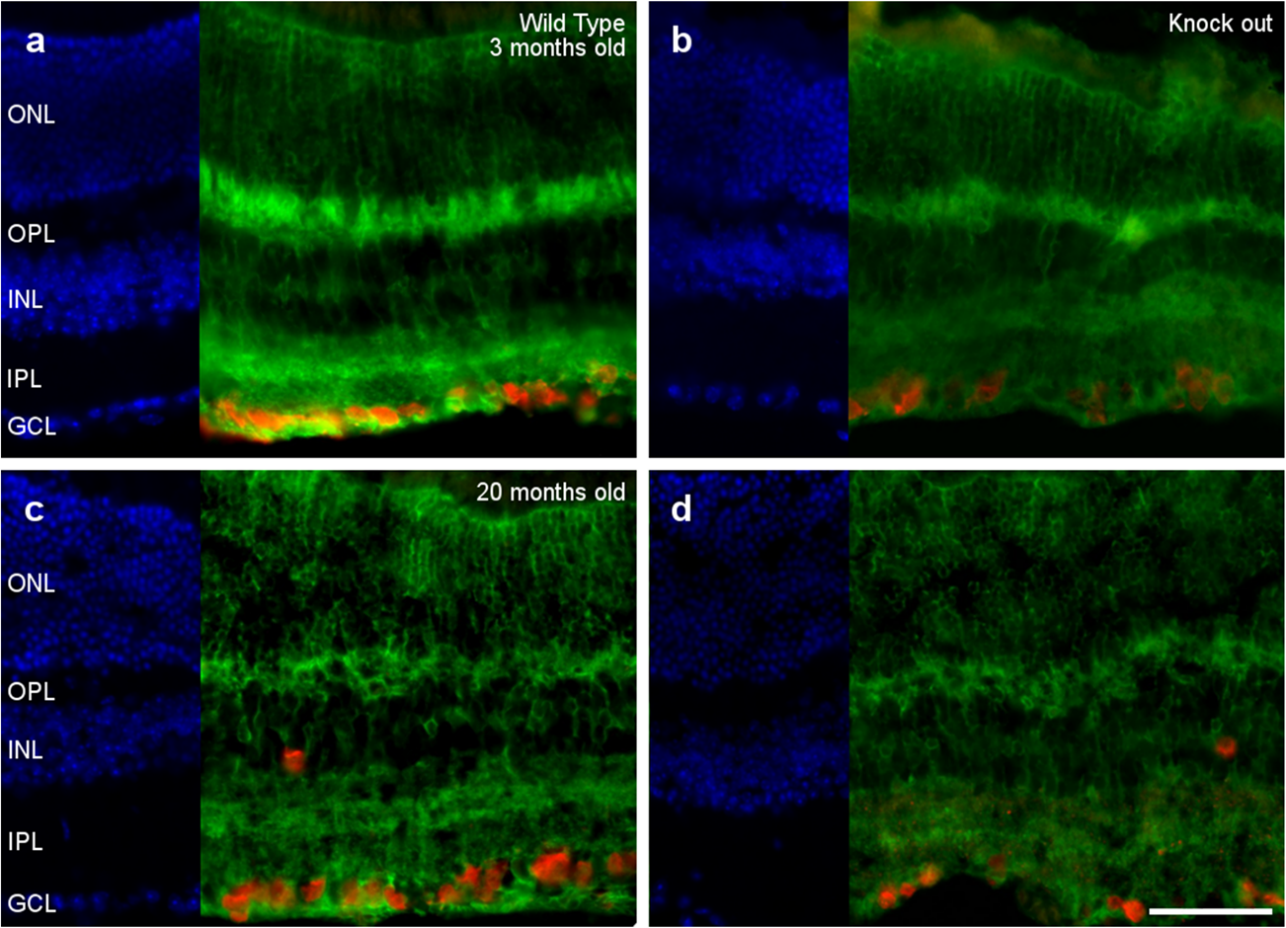
Müller cell analysis. Retinal sections from 3- (a, b) and 20-month-old (c, d) wild type (a, c) and OPN knock-out (b, d) mice, in which the nuclei are labeled with DAPI (blue), Müller cells with an antibody against glutamine synthetase (green), and the RGCs are labeled with antibody against RBPMS (red): ONL, outer nuclear layer; OPL, outer plexiform layer; INL, inner nuclear layer; IPL, inner nuclear layer; GCL, ganglion cell layer. Scale bar = 50 μm

Finally, we did not detect differences between the Müller cell structure in wild type and OPN knock-out mice at 3 and 20 months of age (Fig. 5). In both cases, the Müller cells, labeled by the antibody against glutamine synthetase, cross the retina in a radial orientation and their processes surrounded the cell bodies of retinal neurons. Although the morphology of the retina may be altered in 20-month-old mice, the Müller cells continue to surround the soma of the RGCs (*See* Fig. 5) and the photoreceptors (ONL in Fig. 5).

## Discussion

Since OPN is involved in many important homeostatic processes [6–8], the OPN knock-out mouse model offers the possibility of studying how OPN affects the retina. In aging, the expression of OPN in the CNS gradually decreases [32], whereas OPN expression is induced or upregulated in response to damage [20, 23, 29, 39]. This induction of OPN expression is evident in glial cells like astrocytes and microglia [20, 23, 39], and the absence of OPN could alter nervous tissue. In the retina of OPN knock-out mice, the RGC density is reduced, as is the surface occupied by astrocytes.

The spinal cord of these OPN-deficient mice has a percentage of white matter significantly different to that of wild type mice [12], and the mechanical withdrawal threshold increases significantly in OPN knock-out mice [4]. In addition, when the nervous system of these mutant mice is injured, it suffers more damage due to the lack of OPN [6, 12, 40, 41]. The decrease in the number of RGCs in the OPN knock-out mice may reflect the more limited neuroprotection in these animals [42]. OPN has positive effects on survival, proliferation, migration, and neural stem cell differentiation [43], and nasal administration of OPN decreases neuronal cell death and brain edema after insult [44]. In the retina, the neuroprotective effect of OPN has been demonstrated in porcine photoreceptor cells, significantly reducing the proportion of apoptotic cells [30]. Moreover, the neuroprotective effect of OPN has been seen in RGCs after ischemic-like damage [13], and it can stimulate axon growth in RGCs [45]. Conversely, OPN is also significantly correlated with the progressive degree of optic nerve degeneration and RGC loss in a mouse glaucoma model. In fact, OPN treatment inhibited cell degeneration within the ganglion cell layer in cultured glaucomatous eyes [16]. Thus, not all RGCs appear to have the same needs, and some RGCs can survive for almost the entire life of the OPN knock-out mice. However, at least one subpopulation of RGCs appears to need OPN to survive, and thus, these cells could be rescued by OPN.

The neuroprotective properties of OPN might be associated with an integrin-linked kinase and CD44 signaling. Integrin receptors trigger Akt and FAK activation, which stimulates the phosphoinositide 3-kinase pathway that is in turn directly associated with cleaved-caspase-3 inhibition and anti-apoptotic cell death [44]. This has been demonstrated in the developing brain using an integrin antagonist that attenuates the neuroprotective effect of OPN [46]. In addition, OPN treatment of cortical neuron cultures causes an increase in Akt and p42/p44 mitogen-activated protein kinase phosphorylation, again consistent with OPN induced neuroprotection [42]. OPN can also upregulate the phospho-Akt, cyclin D1 and phospho-Rb content of cells, subsequently enhancing the proliferation of neural progenitors in the presence of fibroblast growth factor 2 [47]. Moreover, OPN can also protect against toxicity by decreasing glial cell activation [48].

OPN is also necessary for a subpopulation of astrocytes, as we find that astrocytes are absent from some areas of the retina in the OPN knock-out mice. The reduction in the surface area occupied by astrocytes could be a consequence of OPN’s role in cell adhesion, migration and survival, and its influence on the metabolic activity of astrocytes [1]. The influence of OPN on the survival of astrocytes has been demonstrated in glioblastoma, with OPN secreted by stromal astrocytes conferring them resistance to radiation [49]. In addition, only astrocytes that express OPN survive after ischemic injury in the brain, because OPN is involved in calcium precipitation and it allows astrocytes to participate in the phagocytosis of calcium deposits [50]. Therefore, the lack of OPN could make some subpopulations of astrocytes more sensitive to cell death than others. This astrocyte heterogeneity has also been demonstrated in relation to other issues [18, 51], and these cells could be implicated in different pathways of neuroprotection.

We also found that the distribution of astrocytes and the RGC density in the 3-month-old OPN knock-out mice is similar to that found in 20-month-old wild type mice. This could indicate that the absence of OPN induces premature aging in the retina. In aged rats, there is an increase in GFAP protein, although astrocytes are less numerous and have distorted morphologies, with shorter and fragmented branches [52]. Indeed, the astrocytes in the aging rat retina are very similar in appearance to those found in the OPN knock-out mouse retina [53, 54]. The lack of astrocytes and RGCs is more evident in the peripheral areas of the retina than in the central areas. This is consistent with descriptions that the peripheral retina is more sensitive to damage such as experimental glaucoma, where more RGC death has been described [55–57]. Since the loss of astrocytes is very dramatic in peripheral areas of younger animals (3-month-old animals) and it is maintained along the animal’s life (20-month-old animals), the reduction in the number of RGCs takes place progressively during the animal’s life, indicating that RGC cell death could be secondary to the astrocyte death.

Although there are signs of degeneration in the OPN knock-out retinas, there were no signs of activation in microglial cells, such as migration to the subretinal space from the inner parts of the retina [58] or a change in their morphology to amoeboid microglia [59]. This is consistent with what is seen in the brain, where no sign of microglia activation has been found in OPN-deficient mice [6]. Although the number of microglial cells increases in aged retinas [60], as evident in 20-month-old retinas, there are no differences between knock-out and wild type retinas. Microglia undergo morphological changes with age, with gradually larger cell bodies, as well as progressively shorter and thicker cell processes [36]. However, the microglia in the OPN knock-out retinas do not show signs of aging relative to the wild type retinas. OPN could be synthesized de novo by activated microglia in response to retinal neurodegeneration [13], and OPN can activate amoeboid transformation, phagocytosis, and the motility of the microglia [61]. Thus, the lack of OPN could prevent the shift of microglia to an amoeboid phenotype and there acquisition of migratory capacity.

A characteristic pattern of OPN may be found in Müller cells of control retinas [62] and through its presence in the secretome of Müller cells [31]. Thus, Müller cells can express and secrete OPN in response to GDNF (glial cell-derived neurotrophic factor) and this OPN exerts a direct effect on the survival of photoreceptors, possibly stimulating Müller cells to overexpress other cytokines with neuroprotective activity [30]. The neuroprotective effects of OPN may be in part mediated by preventing cytotoxic Müller cell swelling, as well as by the release of VEGF (vascular endothelial growth factor) and adenosine from Müller cells [63]. There are no apparent changes in the Müller cells of the retina in OPN knock-out and thus, OPN may not be necessary for the survival of Müller cells, although it could affect their response to cell damage.

In OPN knock-out mice, there is a decrease in RGC density and a reduction of the surface area occupied by astrocytes. Peripheral areas of the retina seem to be more sensitive to damage than central areas and these changes become more prominent with age. Moreover, the density of RGCs and astrocytes in the retina of 3-month-old OPN knock-out mice is very similar to that in the 20-month-old wild type mice. These results suggest that the lack of OPN may induce premature aging. However, microglia and Müller cells seem not to be affected by the lack of OPN, at least not when only aging is considered and the retina suffers no damage. Thus, OPN could be a candidate molecule to develop treatments to combat neurodegenerative disease and astrocytes may represent a specific target of interest in such circumstances.

## References

[1] Neumann C, Garreis F, Paulsen F, Hammer CM, Birke MT, Scholz M (2014). Osteopontin is induced by TGF-beta2 and regulates metabolic cell activity in cultured human optic nerve head astrocytes. PLoS One.

[2] Wang KX, Denhardt DT (2008). Osteopontin: role in immune regulation and stress responses. Cytokine Growth Factor Rev.

[3] Giachelli CM, Lombardi D, Johnson RJ, Murry CE, Almeida M (1998). Evidence for a role of osteopontin in macrophage infiltration in response to pathological stimuli in vivo. Am J Pathol.

[4] Marsh BC, Kerr NC, Isles N, Denhardt DT, Wynick D (2007). Osteopontin expression and function within the dorsal root ganglion. Neuroreport.

[5] Denhardt DT, Noda M, O'Regan AW, Pavlin D, Berman JS (2001). Osteopontin as a means to cope with environmental insults: regulation of inflammation, tissue remodeling, and cell survival. J Clin Invest.

[6] Maetzler W, Berg D, Schalamberidze N, Melms A, Schott K, Mueller JC, Liaw L, Gasser T, Nitsch C (2007). Osteopontin is elevated in Parkinson's disease and its absence leads to reduced neurodegeneration in the MPTP model. Neurobiol Dis.

[7] Scatena M, Liaw L, Giachelli CM (2007). Osteopontin: a multifunctional molecule regulating chronic inflammation and vascular disease. Arterioscler Thromb Vasc Biol.

[8] Maetzler W, Berg D, Funke C, Sandmann F, Stunitz H, Maetzler C, Nitsch C (2010). Progressive secondary neurodegeneration and microcalcification co-occur in osteopontin-deficient mice. Am J Pathol.

[9] Boeshore KL, Schreiber RC, Vaccariello SA, Sachs HH, Salazar R, Lee J, Ratan RR, Leahy P, Zigmond RE (2004). Novel changes in gene expression following axotomy of a sympathetic ganglion: a microarray analysis. J Neurobiol.

[10] Ichikawa H, Itota T, Nishitani Y, Torii Y, Inoue K, Sugimoto T (2000). Osteopontin-immunoreactive primary sensory neurons in the rat spinal and trigeminal nervous systems. Brain Res.

[11] Rittling SR, Matsumoto HN, McKee MD, Nanci A, An XR, Novick KE, Kowalski AJ, Noda M, Denhardt DT (1998). Mice lacking osteopontin show normal development and bone structure but display altered osteoclast formation in vitro. J Bone Miner Res.

[12] Hashimoto M, Sun D, Rittling SR, Denhardt DT, Young W (2007). Osteopontin-deficient mice exhibit less inflammation, greater tissue damage, and impaired locomotor recovery from spinal cord injury compared with wild-type controls. J Neurosci.

[13] Chidlow G, Wood JP, Manavis J, Osborne NN, Casson RJ (2008). Expression of osteopontin in the rat retina: effects of excitotoxic and ischemic injuries. Invest Ophthalmol Vis Sci.

[14] Ju WK, Kim KY, Cha JH, Kim IB, Lee MY, Oh SJ, Chung JW, Chun MH (2000). Ganglion cells of the rat retina show osteopontin-like immunoreactivity. Brain Res.

[15] Osborne NN, Casson RJ, Wood JP, Chidlow G, Graham M, Melena J (2004). Retinal ischemia: mechanisms of damage and potential therapeutic strategies. Prog Retin Eye Res.

[16] Birke MT, Neumann C, Birke K, Kremers J, Scholz M (2010). Changes of osteopontin in the aqueous humor of the DBA2/J glaucoma model correlated with optic nerve and RGC degenerations. Invest Ophthalmol Vis Sci.

[17] Chowdhury UR, Jea SY, Oh DJ, Rhee DJ, Fautsch MP (2011). Expression profile of the matricellular protein osteopontin in primary open-angle glaucoma and the normal human eye. Invest Ophthalmol Vis Sci.

[18] Vecino E, Rodriguez FD, Ruzafa N, Pereiro X, Sharma SC (2016). Glia-neuron interactions in the mammalian retina. Prog Retin Eye Res.

[19] Chabas D, Baranzini SE, Mitchell D, Bernard CC, Rittling SR, Denhardt DT, Sobel RA, Lock C, Karpuj M, Pedotti R, Heller R, Oksenberg JR, Steinman L (2001). The influence of the proinflammatory cytokine, osteopontin, on autoimmune demyelinating disease. Science.

[20] Choi JS, Park HJ, Cha JH, Chung JW, Chun MH, Lee MY (2003). Induction and temporal changes of osteopontin mRNA and protein in the brain following systemic lipopolysaccharide injection. J Neuroimmunol.

[21] Jin JK, Na YJ, Moon C, Kim H, Ahn M, Kim YS, Shin T (2006). Increased expression of osteopontin in the brain with scrapie infection. Brain Res.

[22] Chang SW, Kim HI, Kim GH, Park SJ, Kim IB (2016). Increased expression of osteopontin in retinal degeneration induced by blue light-emitting diode exposure in mice. Front Mol Neurosci.

[23] Choi JS, Kim HY, Cha JH, Choi JY, Lee MY (2007). Transient microglial and prolonged astroglial upregulation of osteopontin following transient forebrain ischemia in rats. Brain Res.

[24] Ellison JA, Velier JJ, Spera P, Jonak ZL, Wang X, Barone FC, Feuerstein GZ (1998). Osteopontin and its integrin receptor alpha(v)beta3 are upregulated during formation of the glial scar after focal stroke. Stroke.

[25] Hashimoto M, Koda M, Ino H, Murakami M, Yamazaki M, Moriya H (2003). Upregulation of osteopontin expression in rat spinal cord microglia after traumatic injury. J Neurotrauma.

[26] Kim SY, Choi YS, Choi JS, Cha JH, Kim ON, Lee SB, Chung JW, Chun MH, Lee MY (2002). Osteopontin in kainic acid-induced microglial reactions in the rat brain. Mol Cells.

[27] Shin T, Ahn M, Kim H, Moon C, Kang TY, Lee JM, Sim KB, Hyun JW (2005). Temporal expression of osteopontin and CD44 in rat brains with experimental cryolesions. Brain Res.

[28] Ellison JA, Barone FC, Feuerstein GZ (1999). Matrix remodeling after stroke. De novo expression of matrix proteins and integrin receptors. Ann N Y Acad Sci.

[29] Wang X, Louden C, Yue TL, Ellison JA, Barone FC, Solleveld HA, Feuerstein GZ (1998). Delayed expression of osteopontin after focal stroke in the rat. J Neurosci.

[30] Del Rio P, Irmler M, Arango-Gonzalez B, Favor J, Bobe C, Bartsch U, Vecino E, Beckers J, Hauck SM, Ueffing M (2011). GDNF-induced osteopontin from Muller glial cells promotes photoreceptor survival in the Pde6brd1 mouse model of retinal degeneration. Glia.

[31] von Toerne C, Menzler J, Ly A, Senninger N, Ueffing M, Hauck SM (2014). Identification of a novel neurotrophic factor from primary retinal Muller cells using stable isotope labeling by amino acids in cell culture (SILAC). Mol Cell Proteomics.

[32] Albertsson AM, Zhang X, Leavenworth J, Bi D, Nair S, Qiao L, Hagberg H, Mallard C, Cantor H, Wang X (2014). The effect of osteopontin and osteopontin-derived peptides on preterm brain injury. J Neuroinflammation.

[33] Bonnel S, Mohand-Said S, Sahel JA (2003). The aging of the retina. Exp Gerontol.

[34] Curcio CA, Drucker DN (1993). Retinal ganglion cells in Alzheimer’s disease and aging. Ann Neurol.

[35] Ramirez JM, Ramirez AI, Salazar JJ, de Hoz R, Trivino A (2001). Changes of astrocytes in retinal ageing and age-related macular degeneration. Exp Eye Res.

[36] von Bernhardi R, Eugenin-von Bernhardi L, Eugenin J (2015). Microglial cell dysregulation in brain aging and neurodegeneration. Front Aging Neurosci.

[37] Pinar-Sueiro S, Zorrilla Hurtado JA, Veiga-Crespo P, Sharma SC, Vecino E (2013). Neuroprotective effects of topical CB1 agonist WIN 55212-2 on retinal ganglion cells after acute rise in intraocular pressure induced ischemia in rat. Exp Eye Res.

[38] Vecino E, Garcia-Crespo D, Garcia M, Martinez-Millan L, Sharma SC, Carrascal E (2002). Rat retinal ganglion cells co-express brain derived neurotrophic factor (BDNF) and its receptor TrkB. Vis Res.

[39] Iczkiewicz J, Rose S, Jenner P (2007). Osteopontin expression in activated glial cells following mechanical- or toxin-induced nigral dopaminergic cell loss. Exp Neurol.

[40] van Velthoven CT, Heijnen CJ, van Bel F, Kavelaars A (2011). Osteopontin enhances endogenous repair after neonatal hypoxic-ischemic brain injury. Stroke.

[41] Wright MC, Mi R, Connor E, Reed N, Vyas A, Alspalter M, Coppola G, Geschwind DH, Brushart TM, Hoke A (2014). Novel roles for osteopontin and clusterin in peripheral motor and sensory axon regeneration. J Neurosci.

[42] Meller R, Stevens SL, Minami M, Cameron JA, King S, Rosenzweig H, Doyle K, Lessov NS, Simon RP, Stenzel-Poore MP (2005). Neuroprotection by osteopontin in stroke. J Cereb Blood Flow Metab.

[43] Rabenstein M, Hucklenbroich J, Willuweit A, Ladwig A, Fink GR, Schroeter M, Langen KJ, Rueger MA (2015). Osteopontin mediates survival, proliferation and migration of neural stem cells through the chemokine receptor CXCR4. Stem Cell Res Ther.

[44] Topkoru BC, Altay O, Duris K, Krafft PR, Yan J, Zhang JH (2013). Nasal administration of recombinant osteopontin attenuates early brain injury after subarachnoid hemorrhage. Stroke.

[45] Ries A, Goldberg JL, Grimpe B (2007). A novel biological function for CD44 in axon growth of retinal ganglion cells identified by a bioinformatics approach. J Neurochem.

[46] Chen W, Ma Q, Suzuki H, Hartman R, Tang J, Zhang JH (2011). Osteopontin reduced hypoxia-ischemia neonatal brain injury by suppression of apoptosis in a rat pup model. Stroke.

[47] Kalluri HS, Dempsey RJ (2012). Osteopontin increases the proliferation of neural progenitor cells. Int J Dev Neurosci.

[48] Broom L, Jenner P, Rose S (2015). Increased neurotrophic factor levels in ventral mesencephalic cultures do not explain the protective effect of osteopontin and the synthetic 15-mer RGD domain against MPP+ toxicity. Exp Neurol.

[49] Friedmann-Morvinski D, Bhargava V, Gupta S, Verma IM, Subramaniam S (2016). Identification of therapeutic targets for glioblastoma by network analysis. Oncogene.

[50] Park JM, Shin YJ, Kim HL, Cho JM, Lee MY (2012). Sustained expression of osteopontin is closely associated with calcium deposits in the rat hippocampus after transient forebrain ischemia. J Histochem Cytochem.

[51] Chaboub LS, Deneen B (2012). Developmental origins of astrocyte heterogeneity: the final frontier of CNS development. Dev Neurosci.

[52] Cerbai F, Lana D, Nosi D, Petkova-Kirova P, Zecchi S, Brothers HM, Wenk GL, Giovannini MG (2012). The neuron-astrocyte-microglia triad in normal brain ageing and in a model of neuroinflammation in the rat hippocampus. PLoS One.

[53] Diniz DG, Foro CA, Rego CM, Gloria DA, de Oliveira FR, Paes JM, de Sousa AA, Tokuhashi TP, Trindade LS, Turiel MC, Vasconcelos EG, Torres JB, Cunnigham C, Perry VH, Vasconcelos PF, Diniz CW (2010). Environmental impoverishment and aging alter object recognition, spatial learning, and dentate gyrus astrocytes. Eur J Neurosci.

[54] Rodriguez-Arellano JJ, Parpura V, Zorec R, Verkhratsky A (2016). Astrocytes in physiological aging and Alzheimer’s disease. Neuroscience.

[55] Quigley HA (2011). Glaucoma. Lancet.

[56] Ruiz-Ederra J, Garcia M, Hernandez M, Urcola H, Hernandez-Barbachano E, Araiz J, Vecino E (2005). The pig eye as a novel model of glaucoma. Exp Eye Res.

[57] Urcola JH, Hernandez M, Vecino E (2006). Three experimental glaucoma models in rats: comparison of the effects of intraocular pressure elevation on retinal ganglion cell size and death. Exp Eye Res.

[58] Omri S, Behar-Cohen F, de Kozak Y, Sennlaub F, Verissimo LM, Jonet L, Savoldelli M, Omri B, Crisanti P (2011). Microglia/macrophages migrate through retinal epithelium barrier by a transcellular route in diabetic retinopathy: role of PKCzeta in the Goto Kakizaki rat model. Am J Pathol.

[59] Lull ME, Block ML (2010). Microglial activation and chronic neurodegeneration. Neurotherapeutics.

[60] Karlstetter M, Scholz R, Rutar M, Wong WT, Provis JM, Langmann T (2015). Retinal microglia: just bystander or target for therapy?. Prog Retin Eye Res.

[61] Ellert-Miklaszewska A, Wisniewski P, Kijewska M, Gajdanowicz P, Pszczolkowska D, Przanowski P, Dabrowski M, Maleszewska M, Kaminska B (2016) Tumour-processed osteopontin and lactadherin drive the protumorigenic reprogramming of microglia and glioma progression. Oncogene 35(50):6366–6377. doi:10.1038/onc.2016.55 10.1038/onc.2016.55 27041573

[62] Deeg CA, Eberhardt C, Hofmaier F, Amann B, Hauck SM (2011). Osteopontin and fibronectin levels are decreased in vitreous of autoimmune uveitis and retinal expression of both proteins indicates ECM re-modeling. PLoS One.

[63] Wahl V, Vogler S, Grosche A, Pannicke T, Ueffing M, Wiedemann P, Reichenbach A, Hauck SM, Bringmann A (2013). Osteopontin inhibits osmotic swelling of retinal glial (Muller) cells by inducing release of VEGF. Neuroscience.

